# How Does Strategic Human Resource Management Impact on Employee Voice Behavior and Innovation Behavior With Mediating Effect of Psychological Mechanism

**DOI:** 10.3389/fpsyg.2022.920774

**Published:** 2022-06-16

**Authors:** Yunhe Li, Li Zhang, Xin Yan

**Affiliations:** ^1^School of Economics and Management, Shangqiu Normal University, Shangqiu, China; ^2^Siam University, Bangkok, Thailand; ^3^College of Business, City University of Hong Kong, Hong Kong, Hong Kong SAR, China

**Keywords:** innovation behavior, psychological contract, self-efficacy, strategic human resource management, voice behavior

## Abstract

Employees’ voice and innovation behaviors are an important source of organizational competitiveness. Scholars in the field of organizational behaviors have discussed how to increase the willingness of employees to engage in voice and innovation behaviors from a diversity of perspectives. Innovation has always been a strategic goal of organizations. To motivate employees to offer valuable advice and innovative ideas, organizations have to provide various incentive, feedback and supportive programs. Combined with the social exchange and social cognitive theories, this study presents an argument that the effective strategic human resource management can gradually improve the self-efficacy, psychological contract, voice behaviors and innovation behaviors of employees, and further verifies the relationship among them. A sample of 553 employees was used and analyzed *via* structure equation modeling. This study adopted PLS-SEM to verify structural model and examine the mediating effect of psychological mechanism. The results showed that strategic human resource management has a significant and positive impact on self-efficacy, psychological contract, voice behavior and innovation behavior; self-efficacy has a significant and positive impact on psychological contract, voice behavior and innovation behavior; psychological contract only has a significant and positive impact on innovation behavior, but not on voice behavior. Given the above research findings, this study gives some practical implications in the end.

## Introduction

COVID-19 has been a neglected issue for a long time. In recent years, COVID-19 has greatly changed the economy, education, society and business of the human beings, followed by many new life and management modes (Lee et al., 2021; Cao et al., 2022; Zhao et al., 2022). Nicola et al. (2020) considered the influence of COVID-19 on world economy in demand sector and supply sector: in demand perspective, quarantine, income loss, and unemployment can be burden of consumer consumption and business investment; in supply perspective, labor availability and output are reduced (Rena and Mbukanma, 2021). However, in the post-pandemic period, companies continue to strengthen important factors that can increase innovation and recovery, including the role of employee innovation behaviors. To adapt to the ever-changing external environment and improve the decision-making quality and effectiveness of organizations, encouraging employees to give voice and increase innovation behaviors (Pieterse et al., 2010; Kang et al., 2015; Bammens, 2016) is the key to enhancing organizations’ innovation capability, as well as of great significance to organizations’ survival (Burris, 2012). Voice behavior refers to constructive advice given by employees against the current situation and problems facing organizations, and the advice will help organizations change the *status quo*, modify procedures, or set out new solutions to enhance the innovation capability (Morrison, 2011; Burris, 2012; Liang et al., 2012). Moreover, innovation behavior refers to the overall process of behavioral expression where employees find, establish and act out creative ideas about new technologies, processes, skills or products (Anderson et al., 2014; Wang et al., 2015; Bammens, 2016). Therefore, how to encourage employees to give voice and enhance their innovation behaviors has been an important topic that raises concerns of scholars and matters the success and long-term development of organizations (Chang et al., 2013; Jia et al., 2014; Morrison, 2014).

As the voice and innovation behaviors of employees are drawing increasing attentions, many studies in recent years have deeply discussed antecedent variables that affect the voice and innovation behaviors of employees (Burris, 2012; Kang et al., 2015, 2016; Bammens, 2016). Generally speaking, the majority of existing literature has focused on the impact of “individual factors” and “contextual factors” on voice behaviors and innovation behaviors of employees. With regard to individual factors, previous studies found that personality traits (Maynes and Podsakoff, 2014), work attitudes (Liang et al., 2012) and work moods of employees can affect their willingness to give voice and increase innovation behaviors. Contextual factors include organizational support solutions and organizational context (Gu et al., 2017; Hsu and Chen, 2017; Rai and Agarwal, 2018). Based on the social exchange theory, scholars emphasized that employees’ voice behaviors and innovation behaviors will carry out the interaction of “Give and Take” in the principle of reciprocity under the premise of long-term return and trust. Examples of interaction include two-way communication and supervisor leadership, organizational resource and encouragement, organization support and cultural climate, etc. (Hsu and Chen, 2017; Rai and Agarwal, 2018). Combined with the findings of previous studies and the social exchange theory, this study proposes the strategic human resource management (SHRM) as an important factor that motivates employees’ voice behaviors and innovation behaviors, and investigates SHRM’s prediction effect.

From the view of resource building, organizations should plan and implement measures and activities that help employees build internal resources of individuals and prepare for career management for a better development (Vuori et al., 2012). The voice behavior and innovation behavior proposed from the perspective of motivating employees (Morrison, 2011) and the SHRM implemented by organizations help employees build relevant psychological resources in the process of socialization, including affectual resources related to decision making, cognitive resources related to learning, and social contact resources dominated by relationships (Ouweneel et al., 2013). These resources will enable employees to have better adaption results such as the enhancement of work satisfaction and the reduction of turnover intention (Gruman and Saks, 2011). Existing literature shows that SHRM can be regarded as a source of resource building, and the relationship between voice behaviors and innovation behaviors can be achieved through the mediating effect of some cognitive variables, during which the transformation of internal mechanisms is required (Gu et al., 2017) before the formation of actual behaviors (Bammens, 2016). However, there are few research findings about the possible explanatory mediating mechanisms among SHRM, voice behaviors and innovation behaviors. To discuss the mediating mechanisms in a more comprehensive manner (Rai and Agarwal, 2018), this study builds a complete conceptual framework based on the social cognitive theory (SCT), and discusses the psychological attitude or cognitive variables as important antecedents that affect voice behaviors and innovation behaviors (Liang et al., 2012; Zheng et al., 2018). By adopting SHRM, organizations can create a positive, socialized, physical environment and atmosphere, in which employees have more opportunities for interaction and feel the concerns for them from the organization, thus mitigating the sense of anxiety, getting rid of negative moods and regaining positive moods (Kammeyer-Mueller et al., 2013). Besides, the SHRM also allows employees to know the operational rules of organizations and what kind of skills are required. This will be further beneficial for the building of confidence (Gruman and Saks, 2011), i.e., self-efficacy, which can be regarded as the building of cognitive resources (Zheng et al., 2018). Furthermore, as indicated by previous studies, indicators related to innovative behaviors of employees are not only associated with the individual attribute, but also depend on the work context (Bammens, 2016), including dynamic or static individual and organizational attributes (Hsu and Chen, 2017). The psychological perception caused by the work context can be regarded as a psychological contract with the organization. This means that there is an intangible and reciprocal relationship between employees and organizations, and that they believe in each other to follow the terms agreed for such a reciprocal relationship and perform corresponding rights and obligations (Chang et al., 2013). From the view of work resources and work context mentioned in SCT, this study aims to discuss the mediating role of self-efficacy and psychological contract, and investigate their effect on voice behaviors and innovation behaviors.

The effectiveness of SHRM on improving employees’ voice behaviors and innovation behaviors depends on the reciprocity driving force and the informal norms in organizations. According to the social exchange theory, there are also unstated emotional relationships and reciprocal obligations between organizations and employees, except for the formal contractual relationship. Employees will further support organizational practices and make more contributions to organizations when they feel the humanized considerations taken by organizations for them. Few prior studies of SCT have discussed voice behaviors of employees. This study combines SCT with the organizational citizenship behaviors and organizational justice to offer rich insights into the theoretical basis of SCT. This argument indicates the organizational citizenship behavior (OCB) plays a critical role in forming a close tie between organizations and employees. As a result, this study aims to discuss the mediating role of OCB in SHRM, voice behaviors and innovation behaviors.

Based on the above arguments, this study takes the SCT as the research perspective, aims to explore the relationships among SHRM, psychological contract, self-efficacy, voice behavior and innovation behavior during the COVID-19 pandemic. This study provides several contributions as following: (1) Applying SCT to establish complete research framework for innovation management; (2) Exploring the importance of psychological mechanism to verify the effect of OCB on innovation and voice behavior; (3) Increasing more insights and understanding to the impact of COVID-19 pandemic on the SCT and OCB.

## Literature Review and Hypotheses Development

### Innovation Behavior

To gain the competitive advantages brought by innovation, organizations need to consider the organizational innovation while understanding the service innovation of partners (Chang et al., 2013); on this basis, innovation behaviors of employees are extremely important for the success and performance of organizations (Anderson et al., 2014; Kang et al., 2015; Wang et al., 2015; Bammens, 2016). Moreover, in recent years scholars have showed great concerns over how to improve innovation behaviors of employees and teams (Jia et al., 2014; Wang et al., 2015). In other words, the sustainable development of an organization depends on whether its employees have good behavioral expressions and continuous innovation capabilities of creating innovative products and services satisfying consumer demands. How to improve indicators related to innovation behaviors of employees has been a topic widely concerned in studies of many scholars (Kang et al., 2016; Gu et al., 2017; Hsu and Chen, 2017). Scott and Bruce (1994) defined the employee innovation based on the view of course proposed by Kanter (1988). At the very beginning, the individual innovation derives from the formation of cognition and concepts over issues; then, individuals seek funding for his/her creative ideas and try to make his/her supporters ally with each other; finally, individuals will develop creative ideas into an innovative prototype or model, and then commercialize it into mass-produced products or services. The discussion of employee innovation involves the formative stage of creative ideas, while the discussion of innovation behaviors also covers the practice of such creative ideas (Anderson et al., 2014). This study argues that innovation behaviors are actually actions taken by employees for the innovativeness, or innovation activities carried out in a course consisting of multiple stages.

### Voice Behavior

Voice behaviors of employees are regarded as a kind of active behavior that drives the growth of organizations. With the purpose of improving the current situation of organizations, employees may, on the one hand, give constructive advice (Rai and Agarwal, 2018), and on the other hand, give a warning and point out potential problems. Active voice behaviors of employees aim to solve problems, but not only make a criticism (Morrison, 2011, 2014; Burris, 2012; Maynes and Podsakoff, 2014). The willingness of employees to take voice behaviors has always been considered as an important topic in studies of OCB (Liang et al., 2012; Rai and Agarwal, 2018). Morrison (2011) argued that the voice should be distinguished in terms of contents and objects, because new advice or problems are proposed based on different benefits and risk considerations. Therefore, Morrison (2011) defined voice as the constructive behaviors that employees express their ideas, opinions or advice over work-related issues to improve the operation of organizations (Maynes and Podsakoff, 2014; Liu et al., 2017). Liang et al. (2012) divided voice behaviors into promotive voice and prohibitive voice. Specifically, the promotive voice means that employees put up with advice or new ideas for the overall operation of employers or organizations, and its purpose is to challenge the current situation, hoping organizations to have changes in terms of innovation and to focus on the future (Morrison, 2014; Liu et al., 2017). Prohibitive voice means that employees concentrate on current problems of organizations, and procedures or events that may damage benefits of organizations, and its purpose is to alleviate and solve problems immediately to protect organizations from losses. Therefore, no matter what type of voice it is, employees will evaluate the price to pay and possible benefits if the object of voice is their supervisors. They will give voice or select appropriate types of voice only when the benefits are higher than the price to pay (Maynes and Podsakoff, 2014; Morrison, 2014).

### Strategic Human Resource Management

The Strategic Human Resource Management (SHRM) explores the interaction between human resource management and management activities, and the relationship between organizational strategies. It plans and manages the durability and integration of human resources in organizations. SHRM allows the human resource department in an organization to design, arrange and train employees that satisfy the needs of the organization in combination with organizational strategies. The resource-based theory holds that human resources in organizations are the source of organizational strengths, and organizations can select highly capable employees through the general human resource management practices. Organizations can integrate their strategies with human resource management after knowing about external environment, opportunities and threats, which is contributive to the formation of competitive advantages and the cooperation among departments. That’s why he human resource development must be combined with the organizational development. And this is also the argument of a strategy-based thinking. By adopting SURM, organizations are able to combine the human resource management with their strategic planning, enabling organizations to integrate activities in a planned way when utilizing human resources, with the purpose of achieving organizational strategies and improving the organizational performance. When the human resource management is combined with strategies, the supervisor-subordinate relationship will no longer exist, and a macro human resource policy and a human resource utilization plan will be formulated at the organizational level. SHRM can result in better organizational performance when employees are competent, although both general and strategic human resource management can improve the organizational performance.

As stated by the social exchange theory, a good social exchange relationship will take shape between organizations and employees when organizations care about employees and take corresponding actions, which can further motivate employees to form work behaviors and attitudes that are beneficial for organizations (Woodrow and Guest, 2020). Previous studies showed that the human resource management practices consistent with organizational strategies are a key influence factor of the psychological contract (Guest and Conway, 2002; Ali et al., 2020). The SHRM is a set of human resource management practices carried out by organizations to realize their strategic goals (Han et al., 2019). Scholars argued that the successful SHRM not only helps employees optimize skills, improve capabilities and increase knowledge reserves, but also enables them to feel supported by organizations, thus strengthening their psychological contract and facilitating them to achieve organizational goals by virtue of their capabilities and motivations (Kang and Snell, 2009; Hernaus et al., 2019). Moreover, SHRM is more likely to offer employees external incentives because of their hard work (Woodrow and Guest, 2020). Based on the social exchange theory and the principle of reciprocity, more voluntary support and higher remuneration from organizations will lead to more strengthened psychological contract of employees, and further facilitate employees to engage in innovation activities (Jiang et al., 2012). Thus, the psychological contract is a key psychological path for SHRM to influence employee attitudes and behaviors. In line with the previous statements, we postulate the following hypothesis:

*H1: Strategic human resource management has a positive impact on psychological contract.*

It is found that individuals’ cognition of context or psychological description of context may affect the behavioral motivations, behavioral patterns and even behavioral outcomes (Elstad et al., 2011; Midtsundstad, 2011). Self-efficacy is people’s capability judgments of, beliefs in or self-control and self-perception over finishing a task in some cases (Teasdale, 2015). As pointed out by Bandura (2013), an individual may reduce his/her self-efficacy when he/she has doubts on his/her capabilities or fails to obtain a positive response from the organization. Human resource management practices in an organization serve as the personnel management context of the organization, and the psychology and behaviors of employees are affected by the human resource management practices all the time. As a result, human resource management practices play a very important role in the formation and development of employee self-efficacy (Maden, 2015). SHRM not only helps employees optimize skills and enhance capabilities and confidence, but also enables employees to feel supported at work by organizations through a range of human resource management practices (Han et al., 2019). As stated by the social exchange theory, organizational behaviors have a positive effect on the employee self-efficacy when resources offered by organizations satisfy exchange requirements of employees (Elstad et al., 2011). Thus, SHRM has a positive effect on the employee self-efficacy. In line with the previous statement, we postulate the following hypothesis:

*H2: Strategic human resource management has a positive impact on self-efficacy.*

Although the purpose of giving voice is to improve the current situation, employees who give voice are still often identified as troublemakers, because they may offend others, bring about negative consequences, and damage self-images in the process of giving advice for organizational improvement (Ashford et al., 1998; Liang et al., 2012). When an organization carries out SHRM practices, it will be more likely to build a close relationship with internal members and attach importance to goals of the group to which those internal members belong, enabling employees to be committed to achieving the common strategic goals (Morrison, 2011). In other words, employees will work harder for the interests of the organization to which they belong when they feel support from SHRM practices (Kim et al., 2013; Rai and Agarwal, 2018). In addition, affected by SHRM, employees will be more concerned about the strategic goals of organizations, and propose complementary suggestions in the process of achieving goals to maintain organizational stability and facilitate the interests and growth of organizations (Grant and Mayer, 2009). In line with the previous statements, we postulate the following hypothesis:

*H3: Strategic Human Resource Management has a positive impact on voice behavior.*

With regard to the discussion over HR activities and organizational innovation, some scholars in recent years asserted that some specific human resource management practices can indeed become the source of innovation (Gu et al., 2017), bringing a higher innovation performance for organizations; such human resource management practices are identified as “best practices,” “high performance work practices” (Aghazadeh and Seyedian, 2004), “HR bundles” (Boselie and Dietz, 2003), and “one best way.” Scholars also debated that organizations that conduct these practices can gain more innovation ideas and behaviors than those that do not conduct (Bammens, 2016). Thus, all the organizations are suggested to adopt these “best practices.” Bammens (2016) also demonstrated that these highly effective human resource management practices can enhance the work motivation of employees and reduce the possibility of loafing on the job through improving their knowledge, skills and capabilities, thus enabling them to engage in more innovation behaviors and facilitating the organizational performance. In line with the previous statements, we postulate the following hypothesis:

*H4: Strategic human resource management has a positive impact on innovation behavior.*

### Self-Efficacy

One of SHRM’s advantages is that it can maintain the flexibility in a dynamic context. It is impossible for an individual to engage in innovation behaviors if he/she lacks intense beliefs in showing the innovation capability. Employees with high skills and rich experience can achieve a good performance only with the help of excellent self-beliefs or ego-involvement motivations (Fast et al., 2014; Hallak et al., 2018). Self-efficacy has been applied by scholars in many theoretical models, including the models of SCT and SCCT (Lent et al., 2011; Lee et al., 2021). For the analysis of the personal determinants in this interactive causal structure, SCT has made important contributions in the process of cognition, substitution, self-regulation and self-reflectiveness. Three aspects of SCT have received particular attention from organizations, including developing people’s cognitive, social and behavioral abilities through proficiency in modeling, cultivating people’s belief in their abilities so that they make full use of their talents, and enhancing people’s motivation through the target system (Lee et al., 2021; Zhao and Zhou, 2021).

In SCT, self-efficacy is deemed as an important self-regulatory mechanism for individuals’ motivations, performance, attitudes and behaviors, determining whether individuals can achieve their goals. Self-efficacy can be discussed from three dimensions: (1) magnitude, which is the difficulty level of tasks for which an individual is competent as he/she believes; (2) strength, which is the individual’s belief that the magnitude is strong or weak; (3) generality, which is the degree to which the expectation can be generalized under circumstances (Lee et al., 2021). The self-efficacy level will affect the motivation of employees to engage in tasks (Lee et al., 2021); Bandura (1997) declared that self-efficacy can be enhanced through self-observation, self-experience and feedback from others, and the specific approaches include achieving success, observing and learning the ways to success of others, being persuaded or gaining positive feedbacks (Slåtten, 2014; Zheng et al., 2018), being motivated physically or psychologically, and being facilitated in a healthy manner (Brown et al., 2011). An individual would seldom take actions or implement tasks once he/she predicts a failure, showing that the employees’ perception of self-competence will affect their cognition, motivation and performance (Caesens and Stinglhamber, 2014). Therefore, this study considers self-efficacy as an important mediator, and holds that employees will make full use of the SHRM of organizations and present better voice behaviors and innovation behaviors.

In the behavior decision-making process, employees will evaluate a range of factors such as motivations, outcomes, beliefs and capabilities to check whether such factors can satisfy the needs before taking practical actions. They will be more inclined to depend on their capabilities and resources, especially in face of high risks and uncertainties for decision making (Fast et al., 2014). Self-efficacy is individuals’ cognitions and beliefs in achieving goals depending on their own capabilities, as well as a key predictor in the OCB (McAllister et al., 2007). However, few studies have discussed the influence of self-efficacy on employees’ voice behaviors (Burris, 2012). As indicated by some scholars, employees can maximize the efficiency through understanding their own capabilities and carefully evaluating steps required to finish every task, so as to achieve established goals with limited resources (Liang et al., 2012; Liu et al., 2017). In this situation, if the process of performing a task needs to be amended, employees with a high level of self-efficacy will give constructive voice to ensure the task performance (Morrison, 2011). However, confronted with uncertainties in the working environment, employees with a low level of self-efficacy are less competent in making effective decisions, and will thus reduce voice behaviors in a risk-averse mindset (Liu et al., 2017). As such, we conclude that a higher level of self-efficacy helps employees control potential negative results, and take voice behaviors required to complete task goals (Liang et al., 2012). In line with the previous statements, we postulate the following hypothesis:

*H5: Perceived self-efficacy has a positive impact on voice behavior.*

Employees’ perception of self-belief is an important condition for predicting their behavioral expression. Previous research shows that individuals with a high level of self-efficacy will be active in setting task goals to be achieved, and effectively control the time and resources required (Lee et al., 2021). Some scholars have attached their research to the concerns for psychological health, POS (Chin and Rasdi, 2014), and life styles for employees (Lent et al., 2011). However, few scholars have ever had an examination on the general self-efficacy and IB. As argued by Jemini-Gashi et al. (2021), individuals show a lower willingness of providing supports, have limited sources of support, and seldom perceive support from others (Brown et al., 2011; Zheng et al., 2018). As reported by Caesens and Stinglhamber (2014), employees with higher self-efficacy tend to be given with a variety of benefits at work, which allows them to generate more job satisfaction (Slåtten, 2014). It can be inferred that the reduction of employees’ general self-efficacy and IB under job stress is caused by the failure in providing them with required mental support in time (Hallak et al., 2018). Moreover, it also facilitates the generation of unique stressors. By contrast, employees representing higher self-efficacy behave better in innovation. In a word, we postulate the following hypothesis:

*H6: Perceived self-efficacy has a positive impact on innovation behavior.*

### Psychological Contract

Cullinane and Dundon (2006) found that the psychological contract originates from the psychological work contract proposed by Argyris (1960) to describe the power nesting and value belief between organizations and employees. Later, Schein (1978) argued that the psychological contract illustrates the expectations held by organizations and employees for each other, the work plan and payment of remunerations, as well as the relationship of rights and obligations between organizations and employees. Thus, the psychological contract is a term defining the agreement on an exchange relationship between individuals and organizations. In terms of the employment relationship, the psychological contract can be interpreted that employees believe the organization will follow terms agreed for corresponding relationships and perform promised obligations. The psychological contract is the individuals’ belief in terms and conditions in a reciprocal exchange agreement concluded with the other party (Chang et al., 2013). Literature shows that the dimension of psychological contract has been diversely classified based on the research interests of scholars (Chang et al., 2013; Wu and Chen, 2015; Gordon, 2020). The psychological contract is generally divided into two dimensions: transactional psychological contract and relationship psychological contract (Chang et al., 2013), which are the most common types of contracts in the employment relationship (DiFonzo et al., 2020). The relational psychological contract concentrates on the inherent, extensive, flexible, unlimited and uncertain obligation relationship, so it pays more attention to the long-term loyalty, trust and job security while involving the monetized transaction relationship. On the contrary, the transactional psychological contract features explicitness, lack of flexibility, and emphasis on the short-term economic and monetized obligational performance; besides, it is also characterized by a narrow contractual scope, and extremely restricted commitment to work from both sides (DiFonzo et al., 2020). In this study, the relational psychological contract is discussed as the major variable to explore innovation behaviors and voice behaviors of employees from the perspectives of reciprocity and long-term relationship.

The psychological contract illustrates the degree of reciprocity brought about by the exchange relationship between employees and organizations, and such degree of reciprocity is reflected by subsequent behavior feedbacks and performance. Meanwhile, it also indicates the interpersonal trust, and the mutual respect perceived by employees in the organizational context. If an employee has a higher sense of psychological contract, he/she agrees more with the management style and culture of the supervisor and the organization, which will be conducive to reducing the perception of environmental uncertainties and the risk of self-expression (Morrison, 2011). As indicated by scholars, employees and supervisors in the social exchange relationship based on the principle of reciprocity are more likely to have positive communications. Moreover, employees will have a high sense of security when giving friendly and constructive voice (Liang et al., 2012; Liu et al., 2017), and do not have to worry about those negative results – for example, being considered as a trouble-maker and causing damages to personal image and status – brought about by expressing opinions or sharing ideas in groups or organizations (Morrison, 2014). As a result, the high level of positive psychological contract is beneficial to reduce the risk and uncertainty of giving voice, and drives employees to take voice behaviors. In line with the previous statements, we postulate the following hypothesis:

*H7: Psychological contract has a positive impact on voice behavior.*

Innovation behaviors are attributed by some scholars to the scope of work performance (Wang et al., 2015). They have been discussing the relationship between the psychological contract and indicators that are used to measure employees’ work performance (Gardner et al., 2015; Harrington and Lee, 2015; Wu and Chen, 2015). Chang et al. (2013) argued that innovation is a long process, and innovation behaviors are the outcome of individuals’ persistent efforts. According to the principle of reciprocity (Dabos and Rousseau, 2004) in the social exchange theory, employees provide feedbacks based on the expected returns but not on the short-term benefits; in other words, employees will have a high level of psychological contract and will be highly motivated to take innovation behaviors if organizations can provide advantageous working conditions to satisfy the needs of employees, resulting in a favorable organization-employee relationship (Wang et al., 2015; Gu et al., 2017). Moreover, it is also found that the high level of psychological contract of employees is significantly associated with the task performance and innovation behaviors (Gardner et al., 2015; Panaccio et al., 2015). In line with the previous statements, we postulate the following hypothesis:

*H8: Psychological contract has a positive impact on innovation behavior.*

**FIGURE 1:**
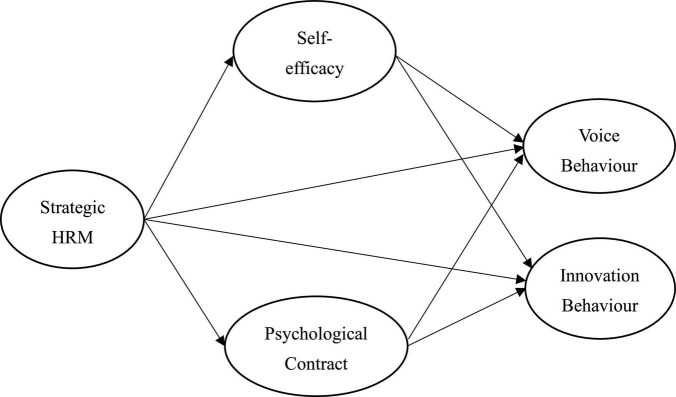
Research framework.

According to above hypotheses, the research framework is shown in Figure 1.

## Methodology

### Sampling

This study aims to understand the impact of SHRM on employees’ self-efficacy, psychological contract, voice behavior and innovation behavior. Different countries are responding and dealing with COVID-19 differently, so it is impracticable to take each country as a sample. This study collects samples from the Chinese mainland. Thus, purposive sampling is conducted to collect data. However, this sampling method has several disadvantages. To avoid these disadvantages, some conditions were set during sampling in this study, which makes the collected samples better conform to the sample reliability and to improves the generalization of the study. This study takes the front-line staff in the service industry, as the study population in order to accurately collect representative samples. In the Chinese mainland, the number of employed persons in the service sector account for 47.4 percent of the total employed population of 770 million, so it is of great practical significance to conduct a study in this sector. In this study, copies of electronic questionnaire were sent, and 582 copies of questionnaire were collected. 553 copies of valid questionnaire were obtained after excluding invalid ones. Most of the organizations represented in the sample were hotels (48%), followed by banks (32%) and other service-based organizations (20%). In total, 59% of the participants were male; 78% employees were being front-level employees classified as staff or specialists and 16% in a mid-level managerial position.

Common method bias (CMB) could be a potential problem in studies that use a single source of data. This study applied “Harman’s single-factor test” to investigate the CMB. The analysis results demonstrate that there are ten factors, of which the explanatory variance of factor 1 is 38.74% that could not explain most of the variance. Thus, there is no common method bias in this study.

This study tested the hypotheses of the research framework and included paths *via* structural equation modeling. Our hypotheses were tested in this study *via* structural equation modeling. In order to verify the construct validity and reliability, confirmatory factor analysis (CFA) was performed using IBM-AMOS 23.0. Finally, partial least squares structural equation modeling (PLS-SEM) was adopted to construct the structural model; specifically, verification of the structural model was performed *via* Smart-PLS 3.0.

### Measures

In this study, we adapt multi-item scale modified from the SHRM Index on seven aspects of SHRM practices proposed by Ali et al. (2018), also well-known as bundle of HR practices. SHRM were measured by seven dimensions, including training (four items, such as “Our organization has formal training activities”), performance appraisal (three items, such as “Results-based appraisal”), staffing (three items, such as “Selection for expertise and skills”), empowerment (four items, such as “Employee has right to take necessary actions on their particular jobs related problems”), promotion (five items, such as “Employees have a well-defined career ladder”), employment security (four items, such as “Employment security plan is discussed with employee before hiring”) and compensation (three items, such as “The link between performance and reward”).

Self-efficacy adopted the scale revised by Alisic and Wiese (2020), and it was revised to integrate three items, such as “My past experiences in my job have prepared me well for my occupational future” and “I can remain calm when facing difficulties in my job because I can rely on my abilities.”

Psychological contract adopted the scale revised by Kraak et al. (2017), which owns six measuring dimensions of job content, career development, social atmosphere, organizational policies, work-life balance and rewards, including 21 items, such as “Offer possibilities for good cooperation,” “Professional development opportunities” and “Clear and fair rules and regulations.”

Voice behavior adopted the scale revised by Wang et al. (2014) based on previous studies, with a total of 6 measuring items, such as “This particular co-worker keeps well informed about issues where his/her opinion might be useful to this work group” and “This particular co-worker communicates his/her opinions about work issues to others in this group even if his/her opinion is different and others in the group disagree with him/her.”

Innovation behavior adopted the scale proposed by Kao et al. (2015) based on previous studies, with a total of 3 measuring items, such as “I often comes up with new and practical ideas to improve performance” and “I often develops new methods for working design.” All Scales were adopted Likert five-point scale which generally used with 1 (strongly disagree) to 5 (strongly agree).

## Results

### Measurement Model

This study uses confirmatory factor analysis (CFA) to measure and also takes into consideration of the criteria of convergent validity set by Hair et al. (2010). As shown in Table 1, Cronbach’s α are from 0.809 to 0.933. What is more, the composite reliability (CR) and average variance extracted (AVE) values of the variables in this study range from 0.899 to 0.955 and 0.706 to 0.900, respectively, and all the variables showed a good fitness, indicating the good convergent validity between the variables in this measurement mode. With regard to discriminant validity, that is, when the square root of the average variance extracted (AVE) is greater than the absolute values of other coefficients relating to the correlation coefficients of this dimension, then the existence of discriminant validity can be supported. The results show that the square root of average variance extracted is greater than the absolute value of any other coefficient on the same column of the Correlation Coefficient Table, so it can be said that this study has discriminant validity.

**TABLE 1:** Measurement.

	1	2	3	4	5	6	7	8	9	10	11	12	13	14	15	16
(1) Training	0.894															
(2) PA	0.708	*0.899*														
(3) Staffing	0.650	0.736	*0.865*													
(4) Empowerment	0.662	0.772	0.806	*0.851*												
(5) Promotion	0.681	0.699	0.748	0.783	*0.872*											
(6) ES	0.632	0.640	0.681	0.719	0.777	*0.840*										
(7) Compensation	0.524	0.628	0.664	0.712	0.777	0.750	*0.867*									
(8) Self-efficacy	0.482	0.444	0.484	0.497	0.546	0.556	0.481	*0.912*								
(9) JC	0.559	0.496	0.544	0.526	0.600	0.589	0.513	0.702	*0.880*							
(10) CD	0.510	0.409	0.463	0.439	0.523	0.520	0.458	0.688	0.798	*0.900*						
(11) SA	0.468	0.326	0.379	0.344	0.455	0.467	0.390	0.652	0.734	0.802	*0.949*					
(12) OP	0.478	0.355	0.409	0.370	0.472	0.509	0.413	0.687	0.753	0.808	0.801	*0.883*				
(13) WLB	0.319	0.311	0.340	0.339	0.369	0.362	0.383	0.513	0.590	0.623	0.629	0.716	*0.849*			
(14) Rewards	0.398	0.272	0.342	0.284	0.389	0.399	0.334	0.609	0.677	0.756	0.771	0.800	0.727	*0.897*		
(15) VB	0.447	0.490	0.489	0.504	0.510	0.491	0.435	0.583	0.532	0.476	0.422	0.445	0.319	0.395	*0.865*	
(16) IB	0.421	0.463	0.450	0.488	0.487	0.469	0.451	0.535	0.491	0.424	0.369	0.380	0.318	0.370	0.760	*0.868*
Mean	3.833	3.604	3.700	3.594	3.774	3.756	3.642	3.971	3.952	4.066	4.127	4.097	3.930	4.126	3.755	3.670
*SD*	0.795	0.803	0.755	0.771	0.757	0.745	0.813	0.683	0.685	0.683	0.719	0.690	0.710	0.702	0.656	0.687
Cronbach’s α	*0.916*	*0.882*	*0.831*	*0.872*	*0.921*	*0.861*	*0.834*	*0.898*	*0.854*	*0.941*	*0.889*	*0.929*	*0.809*	*0.878*	*0.933*	*0.837*
AVE	0.799	0.809	0.748	0.724	0.761	0.706	0.752	0.831	0.774	0.810	0.900	0.779	0.720	0.805	0.748	0.753
CR	0.941	0.927	0.899	0.913	0.941	0.905	0.901	0.937	0.911	0.955	0.947	0.946	0.885	0.925	0.947	0.901

*PA, performance appraisal; ES, employment security; JC, job content; CD, career development; SA, social atmosphere; OP, organizational policies; WLB, work-life balance; VB, voice behavior; IB, innovation behavior.*

*Italicized values mean the squared root of AVE values.*

### Inner Model Analysis

Partial least squares structural equation modeling (PLS-SEM) was adopted to construct the structural model; specifically, verification of the structural model was performed using SmartPLS 3.0 (path analysis). According to Hair et al. (2017), this study assessed the *R*^2^, beta (β) and *t*-value. Their suggestions also emphasized the predictive relevance (*Q*^2^) as well as the effect sizes (*f*^2^). In the structural model, *R*^2^ values obtained for self-efficacy (*R*^2^ = 0.575), psychological contract (*R*^2^ = 0.319), voice behavior (*R*^2^ = 0.416) and innovation behavior (*R*^2^ = 0.368) were larger than 0.3. Prior to hypotheses testing, the values of the variance inflation factor (VIF) were determined. The VIF values were less than 5, ranging from 1.000 to 2.952. Thus, there were no multicollinearity problems among the predictor latent variables (Hair et al., 2017). In the structural model, fit indexes were shown as following: RMSEA = 0.043, SRMR = 0.047, NFI = 0.973.

**FIGURE 2:**
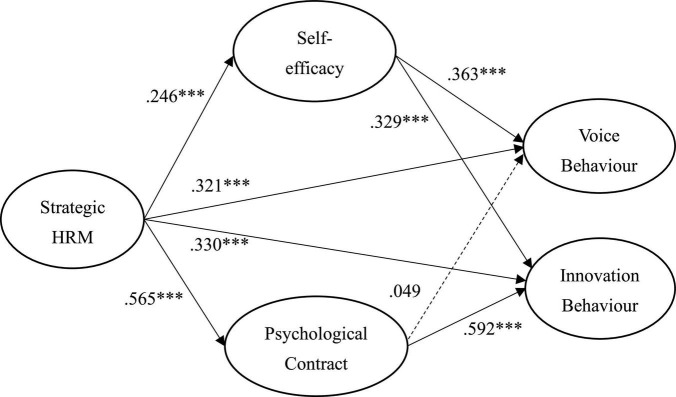
Structural model. ^***^p < 0.001.

Figure 2 and Table 2 shows the results of the hypothesized relationships and standardized coefficients in inner model. The results showed that SHRM was positively and significantly related to self-efficacy (β = 0.246, *f*^2^ = 0.366, *p* < 0.001), psychological contract (β = 0.565, *f*^2^ = 0.366, *p* < 0.001), voice behavior (β = 0.321, *f*^2^ = 0.366, *p* < 0.001) and innovation behavior (β = 0.415, *f*^2^ = 0.366, *p* < 0.001), which supporting H1, H2, H3, and H4. However, self-efficacy (β = 0.330, *f*^2^ = 0.366, *p* < 0.001) was positively and significantly related to voice behavior (β = 0.363, *f*^2^ = 0.366, *p* < 0.001) and innovation behavior (β = 0.329, *f*^2^ = 0.366, *p* < 0.001), supporting H5 and H6. However, our results found that psychological contract (β = 0.392, *f*^2^ = 0113, *p* < 0.001) was positively and significantly related to innovation behavior rather than voice behavior, which only supporting H8 not H7. The Stone–Geisser *Q*^2^ values obtained through the blindfolding procedures for self-efficacy (*Q*^2^ = 0.473), psychological contract (*Q*^2^ = 0.242), voice behavior (*Q*^2^ = 0.306) and innovation behavior (*Q*^2^ = 0.270) were larger than zero, supporting the model has predictive relevance (Hair et al., 2017).

**TABLE 2:** Results of the hypotheses testing.

Paths	Coefficients	*t*-value	Results
H1: SHRM → Self-efficacy	0.246	4.187	Confirmed
H2: SHRM → Psychological contract	0.565	13.726	Confirmed
H3: SHRM → Voice behavior	0.321	5.975	Confirmed
H4: SHRM → Innovation behavior	0.330	6.198	Confirmed
H5: Self-efficacy → Voice behavior	0.363	5.850	Confirmed
H6: Self-efficacy → Innovation behavior	0.329	5.491	Confirmed
H7: Psychological contract → Voice behavior	0.049	0.699	Not confirmed
H8: Psychological contract → Innovation behavior	0.592	9.579	Confirmed

### Examination of Mediating Effects

Self-efficacy and psychological contract in the structural model can be regarded as mediating variables. In order to understand whether self-efficacy and psychological contract have mediating effects, a bootstrapping procedure is further carried out on the structural model. Results displayed in Table 3 indicated that indirect effects of self-efficacy and psychological contract were supported. It shows that the setting of mediating variable plays an important role in the structural model. In particular, self-efficacy, similar to the results of previous studies, can highlight the effects of antecedents in the model, forming strong positive psychology, which are then reflected in the outcome variables.

**TABLE 3:** Indirect effect of structural model.

Paths	Std. β	Std. error	*t*-value
SHRM → Self-efficacy → Voice behavior	0.089***	0.049	6.869
SHRM → Self-efficacy → Innovation behavior	0.081***	0.022	3.668
SHRM → Psychological contract → Voice behavior	0.028	0.039	0.706
SHRM → Psychological contract → Innovation behavior	0.335***	0.047	6.869

***If p < 0.001.

## Conclusion

### Discussion

Voice behaviors and innovation behaviors are important factors for organizations to maintain their growth and stability. This study establishes a research model based on the social exchange theory, and discusses the role and effect of SHRM practices combined with psychological and cognitive factors such as self-efficacy and psychological contract in the social cognitive theory (Brown et al., 2011; Lent et al., 2011; Chin and Rasdi, 2014). The majority of studies focused on the principles of reciprocity and trust in the social exchange relationship (Elstad and Turmo, 2011; Bammens, 2016), but seldom discussed whether SHRM practices can guide employees to take voice behaviors and innovation behaviors through (Bouaziz and Hachicha, 2018). For this reason, this study expects to verify the relationship between variables in combination of different theoretical frameworks, and to offer rich and deep insights into relevant theoretical basis on the grounds of the research results after the verification.

The significance of SHRM has been attested in a majority of studies conducted in western countries (Bouaziz and Hachicha, 2018; Han et al., 2019; Mcclean and Collins, 2019). In particular, organizations in developed countries have regarded SHRM practices as critical organizational behaviors and activities. The research findings show that SHRM has a positive and significant impact on self-efficacy, psychological contract, voice behavior and innovation behavior. This agrees with the conclusion obtained by some scholars (Caesens and Stinglhamber, 2014; Kramar, 2014; Bouaziz and Hachicha, 2018; Rai and Agarwal, 2018). The effective and complete human resource management practices are contributive to increasing the external and psychological resources of employees (Collins, 2021), and achieving the strategic goals of individuals and groups through utilizing such resources (Collings et al., 2021; Collins, 2021). As stated by the social exchange theory, employees will form a psychological cognition that they are concerned and valued in the process when organizations actively adopt the human resource management practices enables (Elstad and Turmo, 2011; Mcclean and Collins, 2019; Gordon, 2020). Drawing on social cognitive theory, Zhao and Zhou (2021) discussed the concept of socially responsible human resource management and empirically examine the impact on hospitality employee’s organizational citizenship behavior for the environment from one chain hotel group. Jiang et al. (2019) explored the dark side of leadership and salespeople’s creativity of pharmacy chain in the context of social cognition and social comparison. Their results indicated that self-efficacy plays an important mediating role. Besides, such practices will be considered as acts of good will that facilitate the establishment of a reciprocal relationship between employees and organizations (Jackson et al., 2014; Bammens, 2016), and will enable employees to be highly motivated to take voice behaviors and innovation behaviors (Bouaziz and Hachicha, 2018). Although SHRM practices can facilitate employees’ voice behaviors and innovation behaviors, it is still necessary to enhance the self-efficacy of employees in order to strengthen their cognition and understanding of task execution and help them engage in effective voice and innovation activities (Caesens and Stinglhamber, 2014). As claimed by the social cognitive theory, the employee self-efficacy plays a vital role in achieving the strategic goals (Bandura, 2013; Chin and Rasdi, 2014), and meanwhile, the overall efficacy of organizations can be elevated while improving the employee self-efficacy (Caesens and Stinglhamber, 2014).

Furthermore, this study assumes that the employee self-efficacy can smooth the way for voice behaviors and innovation behaviors. The results show that the employee self-efficacy has a positive and significant effect on voice behaviors and innovation behaviors. This agrees with the findings of Liang et al. (2012), Caesens and Stinglhamber (2014), Fast et al. (2014), and Hallak et al. (2018) that employees with a higher level of self-efficacy are more willing to provide constructive voice and innovative ideas. This also implies that employees with a better understanding of and a more intense belief in their capabilities are more aware of the way to accomplish tasks and organizational goals efficiently (Bandura, 2013; Hallak et al., 2018). In the context of high task uncertainties, employees will offer a proposal to adjust the process (Hallak et al., 2018), or use new technologies and methods to solve problems, thus achieving organizational goals (Fast et al., 2014; Bammens, 2016). As asserted by models of the self-efficacy theory, social cognitive theory, social cognitive career theory (Chin and Rasdi, 2014), self-efficacy can transform resources of employees and organizations into basic elements required to develop capabilities, and drive employees to develop a belief in achieving goals through the improvement of actual skills, and all these will be reflected by subsequent outputs and behaviors (Caesens and Stinglhamber, 2014).

In this study, it is inferred that the psychological contract can strengthen voice behaviors and innovation behaviors. The findings show that the psychological contract only has a positive and significant effect on innovation behaviors, but not on voice behaviors. As an agreement for the exchange relationship between employees and organizations, the psychological contract is more reflected in the feedback to labor obligations. Employees will be willing to provide more innovative ideas and behaviors when organizations offer higher remunerations to stimulate innovation (Bammens, 2016). This is consistent with the conclusions of Chang et al. (2013), DiFonzo et al. (2020), and Woodrow and Guest (2020). The relationship based on the social exchange relationship will enables employees to pursue for a high level of fairness and justice in organizations (Woodrow and Guest, 2020). Although employees will perform obligations that are proportional to their remuneration, the principle of reciprocity based on the psychological contract will be more helpful to stimulate employees to produce more diversified innovation behaviors through an informal relationship (Hsu and Chen, 2017). However, the psychological contract has no significant effects on voice behaviors, which disagrees with the previous research findings (Liang et al., 2012; Liu et al., 2017). The psychological contract cannot reduce risks and uncertainties brought about by voice behaviors, while it plays an incentive role in employee performance. Employees may think twice and finally choose not to take voice behaviors if they perceive that such behaviors may stir up conflicts or damage the benefits of other (Maynes and Podsakoff, 2014).

### Managerial Implications

The purpose of human resource management is to make the most of capabilities of all employees through a diversity of management practices, thus achieving organizational goals. In this case, inducing all employees in an organization to work for the common organizational goals will be vital for the success of the organization. If organizations want to improve the attitudes (e.g., morale and cohesion) and behaviors (e.g., productivity and turnover) of employees and make full use of resources, the human resource department should set about from the salary and welfare system and the performance evaluation system to guide employees’ behaviors and let them be aware of the internal equity. Besides, since human resource management activities can bring everlasting competitive advantages, the attraction to personnel is also one of the key factors. As such, organizations can attract outstanding personnel by offering remuneration and pay-for-performance programs that are superior to their competitors.

Organizations are suggested to set incentives for giving voice, as a means to reduce the motivation for impression management of employee voice and motivate employees to table critical proposals for organizations. If employees give voice that is helpful to achieve existing goals or find problems from existing process, organizations need to offer incentives such as commendation, bonus and chances of promotion, expecting to transform the motivation into the positive energy that drives organizations forward. In addition, organizations can also use the situational interview during the recruitment to know about whether employees have the personal trait of trying to be the focus, or use tools such as aptitude test to examine whether employees are motivated to find opportunities, bravely face risks and value achievements (Lin and Johnson, 2015).

### Limitations

Since this study is based on cross-industry data, and objective indicators, indicators for voice and innovation behaviors are subjective self-evaluation results. Thus, one of the research limitations is the lack of objective indicators. This study, as a cross-industry one, mainly discusses the effect of SHRM on employees’ voice and innovation behaviors. Objective indicators such as turnover rate and productivity may vary from industries and industrial characteristics, making it difficult to present the objectivity using objective data. That’s why the self-evaluation items are used in this study.

Moreover, this study adopted purposive sampling to collect research participants. Non-random sampling may cause sampling bias. Even a different sampling standard is adopted in this study, the generalization of research findings will also be limited. Thus, researchers are suggested to use random sampling to enhance the representativeness of samples while increasing the number of samples, so as to improve the generalization of research findings.

Furthermore, the long-term effects of SHRM practices are not discussed, because this study is a cross-sectional study in terms of data collection. We suggest carrying out longitudinal studies in the future based on a long-term investigation. In addition, cost consideration is also an elementary factor to be discussed though the high level of SHRM can indeed bring benefits for organizations (Aghazadeh and Seyedian, 2004). However, the cost of SHRM is not considered in this study, leading to the possibility of overestimated effect. For this reason, we suggest future studies should include the cost into consideration when discussing the relationship between the SHRM and the innovation and performance.

## Data Availability Statement

The raw data supporting the conclusions of this article will be made available by the authors, without undue reservation, to any qualified researcher.

## Ethics Statement

The studies involving human participants were reviewed and approved by Academic Committee of School of Economics and Management of Shangqiu Normal University. The patients/participants provided their written informed consent to participate in this study.

## Author Contributions

All authors listed have made a substantial, direct, and intellectual contribution to the work, and approved it for publication.

## Conflict of Interest

The authors declare that the research was conducted in the absence of any commercial or financial relationships that could be construed as a potential conflict of interest.

## Publisher’s Note

All claims expressed in this article are solely those of the authors and do not necessarily represent those of their affiliated organizations, or those of the publisher, the editors and the reviewers. Any product that may be evaluated in this article, or claim that may be made by its manufacturer, is not guaranteed or endorsed by the publisher.
